# Tafenoquine and primaquine do not exhibit clinical neurologic signs associated with central nervous system lesions in the same manner as earlier 8-aminoquinolines

**DOI:** 10.1186/s12936-018-2555-3

**Published:** 2018-11-06

**Authors:** Jonathan Berman, Tracey Brown, Geoffrey Dow, Stephen Toovey

**Affiliations:** 1 Fast-Track Drugs & Biologics, North Potomac, MD 20878 USA; 2 Clinical Network Services Pty Ltd, 88/4 Jephson Road, Toowong, 4066 Queensland Australia; 3 60°Pharmaceuticals LLC, 1025 Connecticut Ave NW, Suite 1000, Washington, DC 20036 USA; 4 Pegasus Research, 4103 Bottmingen, Switzerland

**Keywords:** 8-aminoquinoline, Neurotoxicity, Brain-stem, Neurohistopathology, Malaria, Primaquine, Plasmocid, Pentaquine, Pamaquine, Tafenoquine, Monkeys

## Abstract

**Background:**

Tafenoquine was recently approved for *Plasmodium vivax* radical cure (KRINTAFEL™) and malaria prevention (ARAKODA™).

**Methods:**

A review of the non-clinical and clinical literature was conducted to assess whether tafenoquine (and primaquine) exhibit the same neurologic lesions and associated clinical signs as earlier 8-aminoquinolines, as has been alleged in recent opinion pieces.

**Results:**

Plasmocid, pamaquine and pentaquine damage specific neuro-anatomical structures in Rhesus monkeys and humans leading to corresponding deficits in neurologic function. Neurologic therapeutic indices for these 3 drugs calculated based on monkey data were well correlated with human data. Despite 60 years of use, there is no evidence that primaquine exhibits similar neurotoxicity in humans.

**Discussion/conclusions:**

Extrapolation of data from Rhesus monkeys to humans, and the available clinical data, suggest that tafenoquine also does not exhibit pamaquine, pentaquine or plasmocid-like clinical neurologic signs in humans.

## Background

One of the first 8-aminoquinolines (8AQs) used in clinical practice, plasmocid, was found to cause severe neurologic adverse events at therapeutic doses [1]. Subsequently, a sub-set of these adverse events was also observed in rhesus monkeys and linked to drug-induced degeneration of specific neuro-anatomical structures [2]. Follow-up studies were subsequently conducted in Rhesus monkeys with pentaquine, pamaquine and primaquine (PQ), to determine whether these agents exhibited similar toxicity [3]. Pentaquine and pamaquine were respectively evaluated and commercially used as treatment agents for *Plasmodium vivax*, but were subsequently superseded by the better tolerated primaquine, which has been in clinical use for > 60 years [4]. With tafenoquine now having received regulatory approval in the USA for radical cure of vivax malaria and malaria prevention [5, 6], it is worth considering whether the specific pattern of neurotoxicity observed for plasmocid, pamaquine and pentaquine is associated with this new 8AQ. Such an assessment was made by: (i) comparison of therapeutic indices for neurotoxicity in humans and Rhesus monkeys; and, (ii) query of the safety database submitted by the regulatory sponsor for tafenoquine for malaria prophylaxis using Medical Dictionary for Regulatory Activities (MedDRA) preferred terms to search for specific neurologic signs or symptoms (described later in this review).

For the purposes of this review, neurotoxicity was defined as the observance of specific clinical signs or symptoms (detailed later in this paper), in both Rhesus monkeys and man, which specifically correlate with damage to neuro-anatomical structures observed in rhesus monkeys following administration of 8AQs. For convenience, the phrase ‘neuropathological effect’ is sometimes used as a synonym of ‘damage to neuro-anatomical structures’ and both these phrases are used to cover the full spectrum of damage observed.

The authors acknowledge that the definition of neurotoxicity utilized above could be broadened to address additional issues including: (i) mild-moderate neuropsychiatric events such as insomnia, abnormal dreams, anxiety, and depression in humans [7]; (ii) permanent neurologic lesions observed in rats [8]; and, (iii) rare, but serious psychiatric events such as psychosis in humans [reviewed in 9] associated with mefloquine administration. These other toxicities do not have an obvious correlate of toxicity in the legacy monkey studies. Furthermore, these mefloquine-related issues are beyond the scope of the current review, have been addressed elsewhere or will be subject of dedicated future reviews, but some comments are made regarding these mefloquine-related issues in the next paragraph.

USA prescribing information for tafenoquine for malaria prophylaxis notes a 1% increase in mild-moderate insomnia and depression relative to placebo amongst pooled clinical trial data, in which some of the studies excluded individuals with prior psychiatric history [10]. Furthermore, some of the authors of this paper concluded in a separate publication that the overall adverse event rate of the approved dose of tafenoquine was similar to placebo in a resident population not exposed to the stress of deployment under warlike conditions [11]. The authors acknowledge that mefloquine induces permanent brain stem injury in rats [8] but note that an independent inquiry has determined that there is no evidence that such lesions relate to the neuropsychiatric effects of mefloquine in humans [12]. In contrast, permanent brain stem injury is not seen with tafenoquine in rats at doses that result in plasma levels up to 10-fold higher than those that are clinically relevant [13, 14]. USA prescribing information for tafenoquine for prophylaxis includes a contra-indication for active psychosis as this adverse outcome was observed in 3 individuals with a prior history of this condition who took doses of tafenoquine different from the approved regimen [10]. It is unlikely that all 3 of these cases were adverse drug reactions since the vast majority of individuals with psychosis relapse (and may have relapsed anyway without taking tafenoquine) [15–17]. The label further notes that 3181 individuals (3184 including the 3 mentioned above) received tafenoquine at various doses during the ARAKODA development programme [10], > 1900 of whom were enrolled in clinical trials without specific psychiatric exclusions [18] and > 2000 of whom received doses equivalent to or higher than the approved dose [19]. None of these 3181 individuals experienced psychosis.

For the purpose of this review, neurologic therapeutic indices (neurotoxic dose/antiparasitic dose) were calculated only for a single animal species: Rhesus monkeys. This is because: (i) the archetypal compound, plasmocid, although being neurotoxic in Rhesus, rats and dogs, displayed its most pronounced effects in Rhesus monkeys ([1], see Table 1); (ii) comparable data from other species was not available for pamaquine, pentaquine and PQ; and, (iii) there is no animal model other than the *Plasmodium cynomolgi*-infected Rhesus able to predict efficacy against *P. vivax* hypnozoites in humans [20].

**Table 1:** Neurologic toxicity of plasmocid in different animal species [1]

Species	Minimum lethal daily dose (mg/kg/day)/Minimum cumulative dose causing neurologic signs or lesions (mg/kg)^a^	Human equivalent doses based on body surface area (mg/kg)^b^
Rhesus monkeys	4.5/3	1.4/0.96
Cynomolgus monkeys	< 3/6	< 0.96/1.9
Sooty Mangabey monkeys	24/72	7.7/23
Dogs	3/9	1.7/5
Rats	24/196	3.9/32
Mice	72/144	5.9/12

^a^Doses administered as three equal divided doses daily until animals succumbed or were sacrificed

^b^Calculated according to FDA recommendations [21]

A species-specific and drug dose-based therapeutic index (TI) was utilized rather than a drug exposure-based TI because: (i) the metabolites causing pharmacodynamic effects are unknown for 8AQs; and, (ii) dose-based TIs are what is available in the literature [1–3]. The authors acknowledge that one weakness of this approach is that the safety window for a new 8AQ might be overestimated if the increase in systemic exposure of the new drug relative to increasing dose was higher than that of the benchmark compound (although this does not seem to be the case for tafenoquine).

## Literature search strategy

Pubmed searches were conducted for English language publications prior to 31 December, 2017, using the following collection of search terms: “8-aminoquinoline AND Neurotoxicity”, “Plasmocid”, “Pentaquine”, “Isopentaquine”, “Pamaquine”, “Primaquine and Neurotoxicity”, “Primaquine and Toxicity” and “(8-aminoquinoline OR primaquine) and Rhesus”, (neurotoxicity or toxicity). The subsequent list of publications was screened by one of the authors to identify a list of clinical or non-clinical investigations that might include characterizations of the neurotoxic effects of 8AQs. The short-list of publications was reviewed by a majority of the authors, and the specific references to include in this review were agreed by consensus. Additional studies were added to the list by consensus as needed to provide appropriate background or as sources for additional information such as minimum effective or toxic doses.

## Structures 8AQs included in this review

**Fig. 1:**
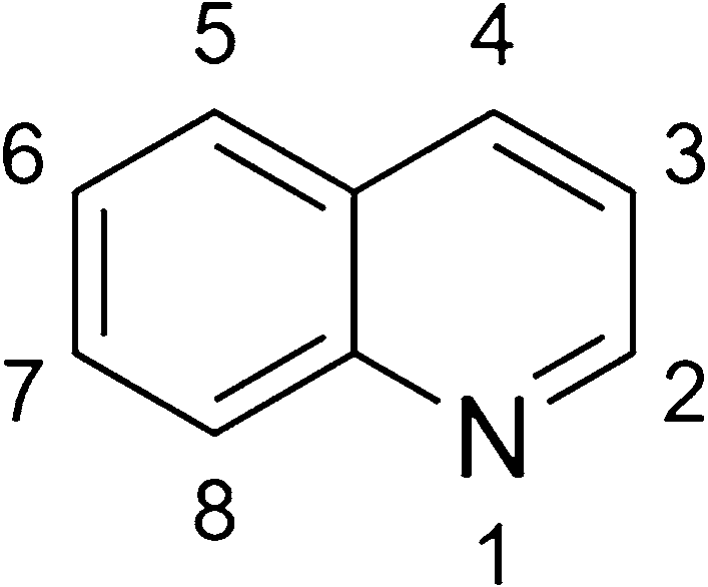
Quinoline numbering system

**Fig. 2:**
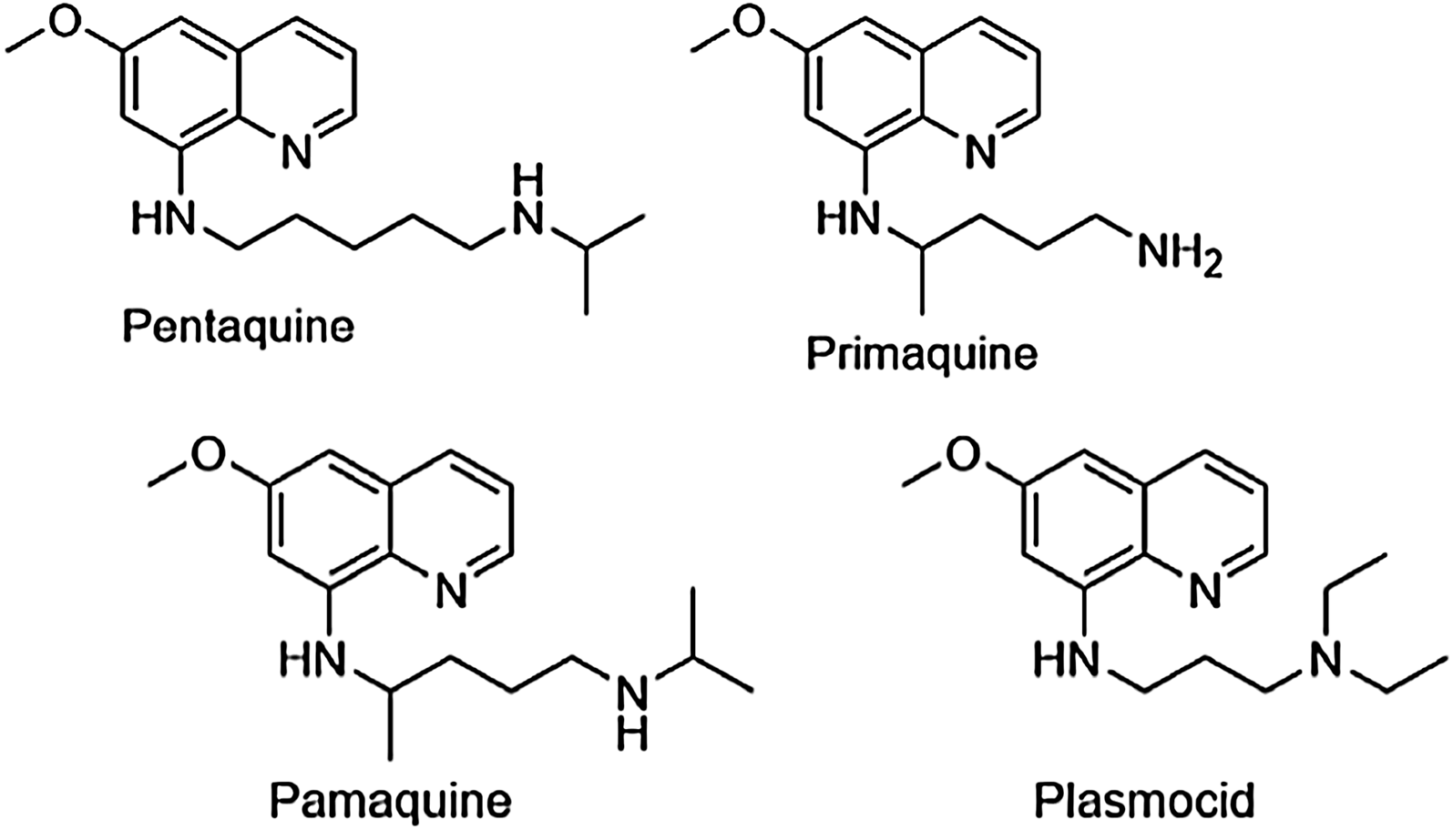
Structures of 8AQs evaluated in this review

**Fig. 3:**
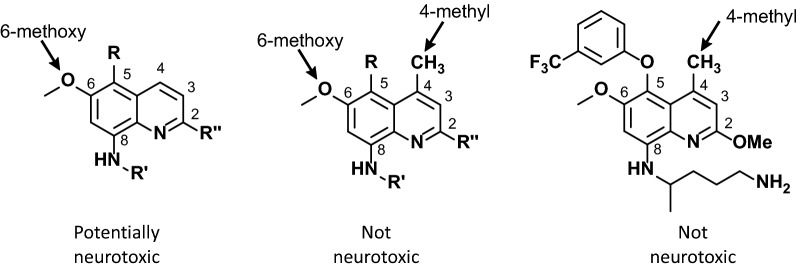
Structure-neurotoxicity relationships for 8AQs and tafenoquine. Tafenoquine is the structure on the far-right

Six 8AQs are referenced in this review. Quinolines are heterocyclic structures containing nitrogen at the first position (Fig. 1). 8AQs are quinolines with an alkylamino side chain at the 8-position and no *N*-alkylamino or *N*-aminoalcohol side chain at the 4-position (Fig. 2). The side chain of primaquine at the 8 position is “(4-amino-1-methylbutyl)amino” (Fig. 2). For PQ the only other substituent on the quinoline ring is a methoxy group at the 6-position. The older 8AQs, pentaquine, pamaquine and plasmocid, differ from PQ only in the side chain at the 8-position (Fig. 2). Tafenoquine retains all of the core structure of PQ, but contains additional ring substitutions including a 4-methyl group (Fig. 3).

## Neurologic toxicity of 8AQs in Rhesus monkeys

Following the observation that plasmocid, an 8AQ synthesized in the 1930s, exhibited clinical neurotoxicity in humans, Schmidt and colleagues [1] gave progressively higher doses of different 8AQs to Rhesus monkeys and evaluated their clinical neurologic effects and neuropathologic effects on the brain. Rhesus monkeys were selected for this purpose as this is the non-clinical species most sensitive to plasmocid-induced neurotoxicity ([1], see Table 1). Rhesus monkeys are also the only non-clinical species in which the radical cure of relapsing malaria parasites can be evaluated [20]. The studies were small, lacked pharmacokinetic data, and did not utilize sophisticated behavioural endpoints as might be employed in a modern safety pharmacology battery. However, they did include specific and detailed neuropathologic investigations and close clinical observation, sufficient to detect neurologic clinical signs previously observed in humans with the same test agents.

8AQs for which there are clinical data and for which published data are available from Rhesus monkeys include pamaquine, pentaquine and PQ. Pamaquine, used before the discovery of PQ, was customarily given at 10–20 mg doses 3 times a day for 5 days [22]. Pentaquine and isopentaquine were the subject of considerable investigation after World War II before PQ was discovered. Primaquine was selected over pamaquine based on their clinical therapeutic indices [“largest (GI) tolerated dose divided by the smallest dose capable of preventing nearly all relapses”]: 10 for PQ, one for pamaquine [23]. The narrative that follows describes the publicly available neuropathological, clinical neurologic and general toxic effects of different 8AQs in Rhesus monkeys. Table 2 presents tabular summaries of doses causing neurologic toxicity, time point of onset of neurotoxicity, doses causing non-neurologic toxicity, therapeutic doses, and therapeutic indices of these 8AQs.

**Table 2:** Neurologic toxicity of 8AQs in Rhesus monkeys

8AQ	MND (mg/kg)	Clinical neurologic signs	Onset of neurologic symptoms (Days)	Dose-limiting toxicity	MED (CD90 in mg/kg)	Neurologic therapeutic index (NTI)
Primaquine	168	None	NA	Malaise, weight loss, leucopenia, hepatotoxicity, methemoglobinemia	7.7	21
Pamaquine	60	None	≤ 7	Cyanosis, methemoglobinemia	14	4.3
Pentaquine	168	Postural hypotension	≤ 12	Cyanosis, methemoglobinemia	~ 45	3.7
Plasmocid	6	Nystagmus, loss of pupillary reflex, equilibrium and motor control	≤ 2	Neurotoxicity^a^	Assume 7.7	< 1

Data from [1–3, 22]

MED refers to the minimum effective dose for radical cure of *P. cynomolgi* in Rhesus monkeys in combination with a blood schizonticide

All doses are the cumulative dose, i.e. the mg/kg/day × number of days dosed

*MND* the minimum cumulative neurotoxic dose causing neurodegeneration or neurologic clinical signs, *NA* not appilicable, *NTI* neurologic therapeutic index

NTI = MND/MED

^a^Same neurologic signs as outlined in Column 3

## Primaquine

For PQ, the lowest cumulative dose tested was 12 mg/kg/day administered daily for 9 days (108 mg/kg) or for 14 days (168 mg/kg) in 2 animals (n = 1/treatment) [3]. At this daily dose, systemic toxicity preceded any loss of neurons. One of the animals died after 9 days of treatment but no loss of neurons was observed. The second animal was sacrificed on day 15 and some loss of neurons was observed. Clinical signs included malaise, methaemoglobinaemia, weight loss, leukopaenia, and hepatotoxicity. At a higher daily dose of 24 mg/kg/day (6 or 14 days), there were signs of degeneration of the dorsal motor, supraoptic and paraventricular nuclei with observation of slight cell loss, slight to moderate pyknosis and moderate to advanced chromatolysis [3]. There were no clinical neurologic signs observed at any dose tested [3]. The minimum neurotoxic dose (MND), defined hereafter as the minimum cumulative dose causing neurodegeneration or associated clinical neurological signs, was 168 mg/kg.

The minimum effective dose (MED) of PQ in Rhesus monkeys is 7.7 mg/kg [24]. Therefore, the neurologic therapeutic index (NTI) of primaquine, defined here, and throughout the rest of this review, as the MND divided by the MED, was 168 mg/kg divided by 7.7 mg/kg or 21 (see Table 2).

## Pamaquine

For pamaquine, doses of 6 mg/kg/day for up to 21 days (126 mg/kg total dose) did not result in loss of neurons [3]. Higher daily doses (12, 18 or 24 mg/kg/day) for shorter periods of time (5–14 days, 6 days or 2–3 days, respectively), yielding cumulative doses much higher than those required to treat malaria, did result in varying degrees of loss of neurons [3].

The minimum effective radical curative dose in Rhesus monkeys is 14 mg/kg [24]. The NTI was estimated to be 60 mg/kg divided by 14 mg/kg or 4.3 (see Table 2).

## Pentaquine

For pentaquine, 12 mg/kg/day × 14 days or a total cumulative dose of 168 mg/kg (n = 2), a dose level much higher than the therapeutic dose, exhibited minor neuropathological changes in the dorsal motor nucleus [3]. Extending the dosing period even higher above the therapeutic level to 20 days (12 mg/kg/day for 20 days (n = 1) for a total cumulative dose of 240 mg/kg) or giving a higher daily dose (24 mg/kg/day for 8–10 days or 48 mg/kg for 4 days (n = 1/regimen) providing a total cumulative dose of 192 or 240 mg/kg/day) resulted in considerable degeneration of the dorsal motor nuclei, nuclei of the supraoptic/paraventricular/Meynart’s group, trochlear and oculomotor nuclei, and finally the hypoglossal and lateral cuneate nuclei [3]. Postural hypotension, potentially related to these lesions, was observed at total doses of 192 mg/kg and higher during the dosing interval [3]. The maximum cumulative dose at which postural hypotension was not observed was 168 mg/kg (12 mg/kg/day for 14 days), in fact animals were reported to be in good health when sacrificed with only cyanosis noted clinically and low grade methaemoglobinaemia [3]. The MED of pentaquine in Rhesus monkeys is approximately 45 mg/kg [24]. Therefore, the NTI was 168 mg/kg divided by 45 mg/kg or 3.7 (see Table 2).

## Plasmocid

In rhesus monkeys, Schmidt and Schmidt [1, 2] found that plasmocid presented an overlapping but more extensive neuropathological picture from that of pentaquine, primaquine or pamaquine. For plasmocid, doses well below the lethal level produced striking symptoms of central nervous system injury associated with severe lesions in the principal nuclei of the proprioceptive, visual reflex and vestibulo-cerebellar pathways [1, 2]. Clinical signs in Rhesus monkeys were consistent with the general and ocular incoordination suggested by these neurological lesions, since fatal intoxication produced a complex group of neurological symptoms including nystagmus, loss of pupillary reflexes, loss of equilibrium, and incoordination of movements of the arms and legs.

Sipe [25] later showed that the vulnerability of certain brain-stem nuclei in Rhesus monkeys to plasmocid was likely mediated by the effect of the drug on neuronal mitochondria.

Expressed in mathematical terms, the minimum cumulative dose causing such histopathological changes was 6 mg/kg. The minimum cumulative dose causing clinical neurologic signs was 6 mg/kg. In contrast to the other 8AQs, Schmidt [2] does not mention any non-neurologic toxicity being observed with plasmocid even during sub-chronic toxicity studies where the total cumulative administered was 63 mg/kg (dose given was 3 mg/kg/day administered as three divided doses daily for 21 days).

Although MED of plasmocid in rhesus monkeys is not known, if one makes the assumption it is equivalent to primaquine, then the NTI is < 1.

## Summary

In summary, it is apparent from the Rhesus data that non-neurologic toxicity preceded neuropathologic or clinical neurologic signs in all cases except for plasmocid, and that for primaquine, pamaquine and pentaquine, neurologic toxicity occurred only at doses associated with lethal toxicity.

For Rhesus monkeys exposed to 8AQs, Schmidt and Schmidt [3] summarized that administration of:

“either pentaquine, ….. primaquine, or pamaquine (at much higher doses than required for malaria therapy) produced significant injury to specific areas of the brain-stem. One group, involved regularly and affected extensively, included the dorsal motor nucleus of the vagus, the supraoptic and paraventricular nuclei, and a small group of cells associated with Meynert’s commissure. The second group, affected less frequently and to a lesser degree, included the abducens, trochlear, and lateral oculomotor nuclei.”

The functions of these brain-stem nuclei and associated nerves are [26]:

- *Vagal dorsal motor nucleus*: The vagus nerve (10th cranial nerve) parasympathetically regulates the heart, gastrointestinal system, and larynx.
- *Supraoptic and paraventricular nuclei (in the hypothalamus)*: Among other products, the cell bodies produce vasopressin that is an anti-diuretic hormone and increase peripheral vascular resistance and blood pressure.
- *Abducens nucleus*: The abducens nerve (6th cranial nerve) regulates the ability to move the ipsilateral eye outward (abduction).
- *Trochlear nucleus*: The trochlear nerve (4th cranial nerve) innervates the superior oblique muscle of the eye.
- *Lateral oculomotor nucleus*: The oculomotor nerve (3rd cranial nerve) maintains an open eyelid by innervating the levator palpebrae superioris muscle.

If lesions to these nuclei/nerves were to be produced by 8AQ in humans, the lesions would be clinically evident by abnormalities of the heart/(centrally effected) gastrointestinal system/lung, blood pressure, eye movements, and diuresis. For Rhesus monkeys exposed to plasmocid, a different pattern of brain injury was seen: lesions in the principal nuclei of the proprioceptive, visual reflex and vestibulocerebellar pathways. If lesions to these latter nuclei/nerves were to be produced by an 8AQ in humans, the lesions would be clinically evident by general and ocular incoordination.

## 8AQ neurotoxicity in humans

### Neurotoxicity of primaquine at therapeutic doses

In the discussion that follows, a distinction is drawn between signs of neurologic toxicity as described earlier (“correlate to damage to neuroanatomical structures observed in Rhesus monkeys following administration of 8AQs”) and more general ‘neuropsychiatric’ effects. The latter may have a neurologic component, but they may also occur in the absence of underlying structural neurologic damage.

Presently recommended regimens of primaquine for complete elimination of hepatic parasites (*P. vivax*) are 15–30 mg per day for 14 days or 45 mg weekly for 8 weeks [27]. To eliminate *Plasmodium falciparum* gametocytes, one dose of 45 mg is used [28].

The major review by Hill et al. [27] and the primaquine label [29] do not mention neurological side effects. When neurological side effects of primaquine were deliberately investigated in normal volunteers to see if the drug might be a threat to performance of air-force duties, 30 mg daily for 7 days had no significant impact on serial reaction time, logical reasoning, serial subtraction, or multitask performance [31].

The most prominent adverse effects in glucose-6-phosphate dehydrogenase (G6PD)-normal individuals are gastrointestinal. Given the possible effects of 8AQ, including primaquine, on the vagal nerve suggested by the Rhesus monkey studies, it is important to note that gastrointestinal distress due to primaquine is local and not central in origin. Tellingly, the gastrointestinal symptoms associated with primaquine are ameliorated by food, despite substantially greater absorption of the drug in the fed versus fasted state [26]. Interestingly, primaquine can also be given for up to year at a dose twice as high as the labelled dose and exhibits a similar tolerability profile to placebo [30–32].

The World Health Organization recently conducted a systematic review of literature and described the safety of primaquine throughout the 60+ years of its use [4]. A single case of depression and a report of an unspecified number of transitory neurologic problems following mass drug treatment are mentioned. Three severe adverse psychiatric events were reported to the Uppsala Monitoring Centre [4], however these are unlikely to be genuine primaquine-attributable adverse drug reactions since they were confounded by mefloquine co-administration.

Finally, in a meta-analysis of adverse event data from 51 studies involving quinoline and non-quinoline anti-malarials, primaquine at the labelled dose exhibited amongst the lowest risk of general neurologic and neuropsychiatric adverse events of all the anti-malarials evaluated [33].

### Toxicity of primaquine at higher doses

Despite the statement in the PQ label [29] that symptoms of over-dosage include neurologic effects (“abdominal cramps, vomiting, burning epigastric distress, central nervous system and cardiovascular disturbances, cyanosis, methaemoglobinaemia, moderate leukocytosis or leukopaenia, and anaemia”), review of the literature in which large doses of PQ have been given do not mention neurologic adverse reactions. In challenge studies, primaquine was administered to 7 volunteers at 60 mg daily for 14 days and to 11 volunteers at 120 mg daily for 14 days. There is no mention of neurologic side effects [3]. Three patients received 240 mg daily for 14 days, a dose that is fully 16-times the 15-mg labelled dose and 8 times the 30-mg suggested to be required to treat Chesson strain *P. vivax* [27], again with gastrointestinal side effects being prominent but no mention of neurologic effects [34].

Therefore, the literature suggests that primaquine, at doses up to 16× the labelled dose in humans, does not cause the neurologic events associated with other earlier 8AQs in humans and monkeys (see Tables 2 and 3).

**Table 3:** Neurologic toxicities of 8AQs in humans

8AQ^d^	Minimum dose known to be required to induce clinical signs of neurotoxicity in humans^a^	Clinical neurologic signs at minimum neurotoxic dose	Onset of neurologic symptoms (days)	Dose-limiting toxicity	Therapeutic dose in humans^a^, mg/day (total mg)	Neurologic therapeutic index (NTI)^e^
Primaquine [27, 34]	> 240 (> 3360)	None	NA	GI distress	15 (210)	> 16
Pamaquine [35]	1200 (1200)^b^	Paralyzed palate/death	≤ 7	GI distress	30 (150)	8
Pentaquine [40]	120 (1680)	Syncope, postural hypotension, erectile dysfunction	≤ 28	GI distress	60 (840)	2
Plasmocid [1, 2]	Not known	Disturbances in eye movement, muscle and equilibrium control	≤ 2	Neurotoxicity^c^	Not known	NA

^a^Expressed as daily dose in mg with total dose in mg in brackets. For primaquine, pentaquine, and pamaquine this is the dose administered for radical cure of *P. vivax* malaria in combination with a blood schizonticidal drug

^b^Both clinical neurologic and histopathologic changes (at autopsy) were observed at this dose which was mistakenly consumed on a single day

^c^Same symptoms as presented in Column 3

^d^Reference source of data is indicated in square brackets

^e^NTI = Minimum dose known to induce clinical signs of neurotoxicity/therapeutic dose

## Pamaquine

The standard daily dose of pamaquine was either 10 or 20 mg 3 times a day [35]. West and Henderson as described by Loken [35], noted adverse events in 24 of 846 malaria patients (3%) who were treated with 10 mg of pamaquine 3 times a day for 5 days. The symptoms which appeared after the administration 60–150 mg of pamaquine treatment consisted of headache, dizziness, abdominal pains, nausea, vomiting, jaundice, and slight fever. In 2 of these malaria-infected patients, psychosis developed, and in a third case, coma. Generally, these effects are consistent with a diagnosis of malaria [36].

In a different study, Hardgrove, as described by Loken [35], reported experience with 4361 patients administered pamaquine, 10 mg 3 times a day for 5 days, of whom 258 (6%) were admitted to the hospital with pamaquine toxicity after being administered 60–140 mg. The most common symptoms were abdominal pain (69% of 258), dark urine (56%), anorexia (45%), jaundice (45%), headache (39%), nausea and vomiting (34%), feverishness (25%), weakness and malaise (22%), and backache (22%). Less common complaints were vertigo (7%), chest pain (5%), diarrhoea (4%), chills (3%), nasal congestion (3%), cyanosis (2%), photophobia (2%), dysuria (2%), palpitation (1%), prostration (1%), syncope (1%), and anuria (1%). Generally, these effects are consistent with either the commonly observed non-neurologic adverse effects of 8AQs or a diagnosis of malaria [27, 36].

Loken [35] reviews the pamaquine literature and reports one case of death and neurotoxicity due to a pamaquine overdose; Blackie [37] reported one fatal case at the recommended clinical dose in 1935, in which only cyanosis and kidney disease were noted; Cordes [38] reported 2 cases with normal dosing in 1928, one in detail in which brain examination specifically showed nothing noteworthy. In a February 1935 review, Schulemann [39] observed that 20 fatalities had been reported in the literature for pamaquine (“plasmochin”) in clinical use up to that date, with all of these cases showing symptoms of acute haemolysis, similar to blackwater fever, but with no reported observations consistent with neurotoxicity.

The patient described by Loken [35] received 1200 mg pamaquine on 1 day (20–40 times the generally recommended dose: 9-fold higher than the cumulative therapeutic dose) and died 7 days later. The patient had been methemoglobinemic/cyanotic, with difficulty breathing, blurred vision and with a paralyzed palate. Histopathologic examination of the brain of this patient revealed that numerous nerve cells of the nuclei pontis had disappeared and others were in various stages of degeneration. There were no apparent vascular changes. Examination of the remainder of the brain disclosed the following: oedema, perivascular in location, was prominent in many parts of the brain, especially in the white matter. In the oculomotor, trochlear and abducent nuclei there was considerable dropping out of nerve cells, degenerative changes in many that remained, and moderate proliferation of microglia and oligodendroglia. The neurologic changes were so specific as to be unlikely due to hypoxia secondary to methemoglobinemia.

Since corticopontine fibres to cranial nerves V and XII descend to pontine nuclei, injury to these fibres/nuclei would be expected to result in jaw weakness (cranial nerve V) and tongue weakness (cranial nerve XII), but these symptoms were not specifically noted in this clinical report. Loken did mention the similarity between the specific damage to the oculomotor, trochlear and abducens nuclei in his patient (which could have been the cause of the blurred vision) and vestibular nuclei and the similarly specific damage to these nuclei in the Rhesus reports.

In summary, for standard doses, pamaquine was poorly tolerated in 3–6% of patients. In patients who did not tolerate the drug well, nausea and vomiting occurred frequently (34% of the time). Since nausea and vomiting can be associated with vertigo, it is difficult to determine if the less frequent complaint of vertigo (3–5% of patients with toxicity; 0.25% of all patients) was linked to these gastrointestinal effects, had an independent (central neurological) etiology, or was attributable to malaria. In one patient who received 9 times the cumulative recommended dose of drug, injury to the cranial nuclei controlling optical movements occurred and may be the explanation for the blurred vision pre-mortem.

The lack of attributable neurotoxicity with standard doses coupled with the rarity of overdose reports (1 reported case who received 9 times the standard dose) indicates that the recommended doses of pamaquine cannot be concluded to be neurotoxic.

## Pentaquine

Alving et al. [40] reported that 60 mg pentaquine administered once per day for 14 days in combination with quinine prevented approximately 96% of relapses by *P. vivax* Chesson strain malaria. Adverse events reported at this dose were typical of those reported for other 8AQs, i.e., epigastric distress, diarrhoea, vomiting, nausea, headache, and methemoglobinemia.

In a subsequent study, 3 of 5 *P. vivax* patients administered doses twice as high (120 mg per day for 14 days) experienced postural hypotension that persisted for many months with no known cause [41]. Schmidt and Schmidt [3] speculated that these cases were similar to pentaquine-induced neurotoxicity in rhesus monkeys, i.e., the hypotension represents a clinical effect secondary to a pentaquine-induced degeneration of the dorsal motor nuclei.

In summary, there is clinical evidence of neurotoxicity with pentaquine at doses twice those required for malaria therapy.

## Plasmocid

Descriptions of the neurotoxicity of plasmocid in humans are difficult to locate. However, Schmidt and Schmidt [1] note that

“the symptoms of plasmocid intoxication in the human are quite similar to those observed in Rhesus. Plasmocid was used to a limited extent in Russia as an abortifacient. D. Allan Butler (pers. comm.) who reviewed the literature on this subject found reports of 76 cases in which mild to severe symptoms of central nervous system dysfunction were observed. Many of these reactions, particularly the disturbances in equilibrium and coordination mechanisms, and in eye muscle movements, were identical with those of monkeys.”

## Comparison of the neurotoxicity of 8AQs in Rhesus monkeys and humans

For all 8AQs except plasmocid, neurotoxicity is not the dose-limiting toxicity in humans. Epigastric distress was the dose-limiting toxicity for pentaquine, pamaquine and primaquine. There is no evidence of primaquine causing neurotoxicity as defined herein at the labelled dose despite 60+ years of use, and very little evidence of neurotoxicity if a broader definition is applied [4]. Nevertheless, from case reports it is possible to conclude that clinical neurologic signs for plasmocid and pentaquine, and the neuropathologic signs for pamaquine observed in Rhesus monkeys, were observed in a few humans at high doses, and to calculate a NTI for these compounds in man for these compounds. The NTIs in Rhesus *versus* humans were similar in magnitude and rank order: < 1 for both species for plasmocid, 3.7 and 2 for pentaquine, 4.3 and 8 for pamaquine, and 21 and > 16 for primaquine (compare Tables 2 and 3).

Since the use of a NTI in Rhesus monkeys is predictive of neurologic safety margins for radical cure of *P. vivax*, it would be reasonable to use the same approach to make a ‘go/no-go’ decision regarding the progression of a new 8AQ such as tafenoquine into the clinic; as the species most sensitive to 8AQ-induced neurotoxicity, data from Rhesus monkeys could also be used to guide decisions about the starting dose for first-in-human studies. Furthermore, having made the decision to proceed with clinical development, it would be reasonable to rule out neurologic toxicity at particular dose levels using a ‘fingerprint’ of clinical neurologic signs observed for older 8AQs. Neurologic signs observed following plasmocid, pamaquine and pentaquine administration in humans and Rhesus include loss of pupillary reflex, nystagmus, disturbed eye movements, loss of equilibrium control, loss of motor coordination, death, postural hypotension, syncope, erectile dysfunction, and paralyzed palate. These clinical signs all have corresponding MedDRA-coded terms which can be used to search safety databases.

## Tafenoquine

### Tafenoquine is a primaquine congener and structural features suggest neurotoxicity should have been eliminated

As illustrated in Fig. 3, tafenoquine is a congener of primaquine, an 8AQ (it is not a 4-quinoline methanol like mefloquine). The structure of tafenoquine is identical to primaquine with the exception that three substituents were added to the quinoline ring to block sites of metabolic attack. The effect of these substitutions was to increase the half-life relative to primaquine from 6 h to 14 days [42].

One of these substitutions involved the substitution of a methyl group at the 4-position. Schmidt showed, through comparison of 6 pairs of substituted and unsubstituted 8AQs of several types, that this substituent completely abolished neurotoxicity in Rhesus monkeys [43]. Therefore, a priori, tafenoquine would not be expected to be neurotoxic in Rhesus monkeys or humans.

### Tafenoquine in Rhesus monkeys

A toxicology study was performed in rhesus monkeys prior to the implementation of the clinical program (see Table 4). Groups of 3, 3 and 4 animals were dosed with a total of 12, 24 or 48 mg/kg administered as divided doses of 3, 6 or 12 mg/kg/day for 4 days. Animals were directly observed for 4 h following each dose, and tafenoquine and methemoglobin levels were monitored. No neurologic signs of the kind reported for pamaquine, pentaquine or plasmocid were observed. The dose-limiting adverse effects were gastrointestinal irritation and symptoms and methemoglobinemia. At the highest dose (48 mg/kg) 2 animals died, and the principal diagnosis on necropsy was hepatotoxicity. The brain of one of these animals was available for examination and no pathological findings were noted (Table 4). Later, it was determined that the MED of tafenoquine in Rhesus monkeys for radical cure of *Plasmodium cynomolgi* hypnozoites (in combination with blood schizonticidal drugs) was 1.8 mg/kg [44], far less than even the lowest non-neurotoxic dose, 12 mg/kg (Table 4). The therapeutic index calculated on the basis of dose administered represented an improvement relative to primaquine (> 27 vs 21, see Table 4). The therapeutic index based on exposure was > 11, indicating that systemic exposure approximately 2.5-fold less than expected at the lethal dose. Thus, the conclusion, that tafenoquine has an improved safety margin relative to primaquine, would only be incorrect if systemic levels of primaquine in monkeys were approximately 5-fold or more lower than expected at the minimum neurotoxic dose.

**Table 4:** Neurologic safety windows of various doses of tafenoquine in Rhesus monkeys

Tafenoquine dose administered (mg/kg/)/N	Neurologic signs	Other clinical signs	Neurologic therapeutic index (ratio relative to effective dose based on dose administered)^c^	Cmax (ng/ml)	Neurologic therapeutic index (ratio relative to effective dose based on exposure)	Source
1.8^a^/35	None reported	Not described	NA	~ 50	1	[44]
12^b^/3	None	None	6.7	124	2.5	[19, 20]
24^b^/3	None	Vomiting, methemoglobinemia	13	284	5.7	[19, 20]
48—Non-Lethal^b^/2	None	Methemoglobinemia	27	333	6.7	[19, 20]
48—Lethal^b^/2	No pathological changes in CNS at autopsy	Vomiting, poor appetite, listlessness, depression, death, hepatotoxicity (amongst other findings noted on necropsy)	27	551	11	[19, 20]

*N* number of animals

^a^Administered as three equal divided doses over three days. This is the 95% curative dose of tafenoquine for radical cure of *P. cynomolgi* in Rhesus monkeys in combination with blood schizonticidal drugs

^b^Administered as four equal divided doses over four days

^c^Calculated by dividing the total dose administered in column 1, rows 2, 3 or 4, by the 95% curative dose (1.8 mg/kg) listed in row 1 of column 1

### Tafenoquine does not exhibit neurotoxicity at prophylactic doses in humans

The improved therapeutic index of tafenoquine relative to primaquine in Rhesus monkeys suggests that tafenoquine should not have exhibited the clinical neurologic side effects of earlier 8AQs over the range of doses that would be employed in a Phase 1 development programme (single or multiple doses over a short duration). To test this, the authors coded the neurological symptoms observed following pentaquine, pamaquine or plasmocid administration in monkeys and/or humans into MedDRA-preferred terms to search the safety database submitted to regulatory agencies by the sponsor. The results of the tafenoquine Phase I programme showed no evidence of pamaquine, pentaquine or plasmocid-like neurotoxicity at doses up to 600 mg, and confirmed that, as in monkeys, and like primaquine in humans, the dose-limiting toxicities were gastrointestinal in nature (Table 5 and [42]).

**Table 5:** Clinical neurologic toxicity is not observed following short or long-term dosing in humans [18]

Neurologic symptom associated with plasmocid, pamaquine or pentaquine in rhesus monkeys or humans	MEDRA code	Number of subjects affected (%)				
		Tafenoquine (total dose)				Placebo
		Phase 1 [44] (4–600 mg)	200 mg × 3 (600 mg)	400 mg × 3 (1200 mg)	200 mg × 3 then 200 mg weekly (average duration 21 weeks) (3000 mg)	
		N = 45	N = 491	N = 713	N = 825	N = 396
NystagmusLoss of motor	Nystagmus—10029864	0 (0.0)	0 (0.0)	0 (0.0)	0 (0.0)	0 (0.0)
Coordination	Coordination abnormal—10010947	0 (0.0)	0 (0.0)	0 (0.0)	2 (0.2)^a^	0 (0.0)
Loss of equilibrium	Balance disorder—10049848	0 (0.0)	0 (0.0)	0 (0.0)	0 (0.0)	0 (0.0)
Loss of pupillary reflexes	Pupillary reflex impaired—10037352	0 (0.0)	0 (0.0)	0 (0.0)	0 (0.0)	0 (0.0)
Death	Death—10011906	0 (0.0)	0 (0.0)	0 (0.0)	0 (0.0)	0 (0.0)
Syncope	Syncope—10042772	0 (0.0)	0 (0.0)	0 (0.0)	2 (0.2)^b^	0 (0.0)
Postural hypotension or Hypotension	Postural hypotension or hypotension—10021097 or 10036433	0 (0.0)	0 (0.0)	1 (0.1)	0 (0.0)	0 (0.0)
Erectile dysfunction	Erectile dysfunction—10061461	0 (0.0)	0 (0.0)	0 (0.0)	1 (0.1)	1 (0.3)
Paralyzed palate	Araflexia—1003084	0 (0.0)	0 (0.0)	0 (0.0)	0 (0.0)	0 (0.0)
Eye movements disturbed	Ophthalmoplegia—10030875 or	0 (0.0)	0 (0.0)	0 (0.0)	0 (0.0)	0 (0.0)
	Extraocular muscle paresis—10015829 or	0 (0.0)	0 (0.0)	0 (0.0)	0 (0.0)	0 (0.0)
	Diplopia—10013036	0 (0.0)	0 (0.0)	0 (0.0)	0 (0.0)	0 (0.0)

^a^Both subjects reported abnormal coordination at the beginning of the study (Day 0), suggesting pre-existing factors were at play. One subject had a history of spinal surgery, while the other had been using loratadine for 7 years

^b^In both cases, there was a single episodes of syncope that was considered mild and unrelated to tafenoquine

There is also no evidence of any plasmocid, pentaquine or pamaquine-like neurotoxicity at the intended prophylactic dose of tafenoquine of 200 mg × 3 followed by 200 mg weekly for up to 6 months in phase 2 and phase 3 studies (Table 5). Two tafenoquine-treated subjects were found to have abnormal coordination (Table 5). In both cases, the abnormality was first documented at the very beginning of the study (day 0), suggesting that this adverse event was influenced by pre-existing factors. In one subject, an important confounding factor was the subject’s chronic use of loratadine to treat allergies, which began 7 years prior to study entry and continued throughout the study. Even at a typical 10 mg dose, loratadine can cause motor control side effects [45], and these effects can become even more apparent when the drug is taken on a chronic basis [46, 47]. The second subject had a history of spinal surgery. Also, two single episodes of syncope were reported (Table 5). Both were mild, isolated episodes that were considered unrelated to tafenoquine. One case was ‘treated’ with acetaminophen.

The safety data for the anticipated clinical dose were collected from 5 different clinical studies [11]. One of these studies was the Phase III study in deployed Australian soldiers in which the intended dose was evaluated for 6 months [48]. Another of the studies was a Phase II study, which was conducted prior to the Phase III study, and evaluated the intended dose for a shorter duration (12 vs 24 weeks) and a higher loading dose (400 mg × 3) than that used for the anticipated clinical regimen [49]. The lack of neurologic signal in those two dosing regimens in Phase II is captured in the aggregate data presented in Table 5.

## Conclusions

The 8AQs plasmocid, pentaquine and pamaquine cause neurotoxicity in Rhesus monkeys characterized by degradation of specific neuro-anatomical structures and clinical signs corresponding to such injury. There is a gradation in the therapeutic indices for this effect that is consistent in monkeys and humans: < 1 for plasmocid, 2–4 for pentaquine and 4.3–8 for pamaquine. Moreover, the dose-limiting toxicity in humans and Rhesus monkeys was neurologic in nature only for plasmocid.

For primaquine, in Rhesus monkeys, doses 21-fold or higher than the effective dose in a *P. cynomolgi* model were required to induce neurodegeneration, and these levels of exposure also caused generalized toxicity. In humans, primaquine is not neurotoxic at the labelled dose used for radical cure despite 60+ years of use and has a therapeutic margin in humans of > 16. Practically, such high doses are rarely if ever reached in clinical practice because the dose-limiting toxicity is epigastric distress. In summary, primaquine does not exhibit the specific type of neurotoxicity associated with earlier 8AQs in either Rhesus or humans.

For tafenoquine, in Rhesus monkeys, doses at least 27-fold higher than the 95% curative dose required for radical cure were not associated with clinical neurologic signs or neurodegeneration. In humans, the anticipated prophylactic dose administered for up to 6 months, and two-fold higher doses than the intended loading dose, did not exhibit any of the specific neurologic signs associated with earlier 8AQs. As with other 8AQs, haematologic toxicity and epigastric distress are dose-limiting. In summary, tafenoquine does not appear to exhibit the specific neurologic signs associated with earlier 8AQs at the doses envisaged for malaria prevention.
